# End-expiratory lung volumes as a potential indicator for COVID-19 associated acute respiratory distress syndrome: a retrospective study

**DOI:** 10.1186/s12890-024-03118-2

**Published:** 2024-06-25

**Authors:** Shengyu Hao, Yilin Wei, Yuxian Wang, Yaxiaerjiang Muhetaer, Chujun Zhou, Songjie Qiong, Pan Jiang, Ming Zhong

**Affiliations:** 1 Department of Critical Care Medicine, Zhongshan Hospital, Fudan University, 180 Fenglin Road, Shanghai, China; 2 Shanghai Institute of Infectious Disease and Biosecurity, Fudan University, 130 Dong’an Road, Shanghai, China; 3 Department of Nutrition, Zhongshan Hospital, Fudan University, 180 Fenglin Road, Shanghai, China

**Keywords:** End-expiratory lung volume, COVID-19, Acute respiratory distress syndrome, Mechanical ventilation

## Abstract

**Background:**

End-expiratory lung volume (EELV) has been observed to decrease in acute respiratory distress syndrome (ARDS). Yet, research investigating EELV in patients with COVID-19 associated ARDS (CARDS) remains limited. It is unclear whether EELV could serve as a potential metric for monitoring disease progression and identifying patients with ARDS at increased risk of adverse outcomes.

**Study design and methods:**

This retrospective study included mechanically ventilated patients diagnosed with CARDS during the initial phase of epidemic control in Shanghai. EELV was measured using the nitrogen washout-washin technique within 48 h post-intubation, followed by regular assessments every 3–4 days. Chest CT scans, performed within a 24-hour window around each EELV measurement, were analyzed using AI software. Differences in patient demographics, clinical data, respiratory mechanics, EELV, and chest CT findings were assessed using linear mixed models (LMM).

**Results:**

Out of the 38 patients enrolled, 26.3% survived until discharge from the ICU. In the survivor group, EELV, EELV/predicted body weight (EELV/PBW) and EELV/predicted functional residual capacity (EELV/preFRC) were significantly higher than those in the non-survivor group (survivor group vs. non-survivor group: EELV: 1455 vs. 1162 ml, *P* = 0.049; EELV/PBW: 24.1 vs. 18.5 ml/kg, *P* = 0.011; EELV/preFRC: 0.45 vs. 0.34, *P* = 0.005). Follow-up assessments showed a sustained elevation of EELV/PBW and EELV/preFRC among the survivors. Additionally, EELV exhibited a positive correlation with total lung volume and residual lung volume, while demonstrating a negative correlation with lesion volume determined through chest CT scans analyzed using AI software.

**Conclusion:**

EELV is a useful indicator for assessing disease severity and monitoring the prognosis of patients with CARDS.

**Supplementary Information:**

The online version contains supplementary material available at 10.1186/s12890-024-03118-2.

## Introduction

Severe COVID-19 pneumonia has presented significant challenges for the research and medical communities. Among individuals hospitalized with COVID-19, 15–30% will progress to develop COVID-19 associated acute respiratory distress syndrome (CARDS) [1]. Autopsy studies of patients who succumbed to severe SARS CoV-2 infection reveal the presence of diffuse alveolar damage, accompanied by a higher thrombus burden in the pulmonary capillaries and fibrosing nonspecific interstitial pneumonia. These factors contribute to reduced functional residual capacity (FRC) and severe arterial hypoxemia [2, 3]. Furthermore, the limitations of using oxygen levels as a prognostic indicator for acute respiratory distress syndrome (ARDS) are well-documented [4, 5]. Additionally, the definition of ARDS has a somewhat controversial history, and the COVID-19 pandemic has further complicated the current Berlin definition of ARDS. Researchers have been advocating for and working towards improved criteria and methods for defining ARDS [6, 7]. It should be emphasized that our treatment objective for ARDS patients should not solely focus on improving their oxygen levels or the ratio of arterial oxygen partial pressure to the fraction of inspired oxygen (PaO₂/FiO₂) [8, 9]. Instead, we require effective and non-invasive monitoring methods to track the progression of ARDS, which are crucial for evaluating patient condition and prognosis. FRC, the amount of gas remaining in the lungs after a natural exhalation at atmospheric pressure, serves as a crucial indicator of gas exchange capacity in healthy individuals [10]. End-expiratory lung volume (EELV), which encompasses the cumulative gas volume within intubated patients, incorporates the FRC along with the additional volume introduced by positive end-expiratory pressure (PEEP) [11, 12]. ARDS leads to a substantial decrease in EELV, resulting in higher strain at a given tidal volume (VT). For this reason, bedside EELV measurement may assist in setting ventilation parameters for protect strategies and better monitoring changes in lung injury [13].

Reproducible measurement techniques are essential for bedside use to minimize overdistention and identify which patients may benefit from recruitment strategies. While CT scans and gas-dilution techniques have been validated for lung-volume measurement, their complexity limits their widespread use in clinical settings. Fortunately, ICU ventilators now offer washout/washin techniques using oxygen or nitrogen, making it convenient to measure EELV at the patient’s bedside. Comparisons between EELV measurements (obtained through multiple breath nitrogen washout/washin and helium dilution) and CT scans have consistently demonstrated strong agreements in stable patients, animal models of ARDS, and artificial lungs [12, 14, 15]. However, the investigation of EELV and its variations, as well as their association with the prognosis of patients with CARDS, remains unexplored.

In this study, we monitored the EELV and its changes in patients with CARDS, and performed a correlation analysis with CT scans. Our hypothesis posits that changes in EELV could serve as a valuable indicator of disease progression and a predictive factor for the prognosis of patients with CARDS, in contrast to relying solely on arterial blood gas measurements and CT volume analysis.

## Materials and methods

The study protocol and informed consent forms were reviewed and approved by the ethics board of Zhongshan Hospital affiliated to Fudan University (approval code: B2023-074R).

### Study population

This study included patients admitted to our ICU between December 2022 to March 2023, who had been diagnosed with COVID-19 infection through confirmation via real-time reverse transcriptase-polymerase chain reaction. The inclusion criteria were as follows: (a) COVID-19 cases classified as severe in accordance with the WHO interim guidance, characterized by clinical signs of pneumonia in addition to a respiratory rate (RR) > 30 breaths/min, severe respiratory distress, and/or oxygen saturation (SpO_2_) < 90% on room air [16]. (b) Endotracheal intubation was administered during the patient’s ICU admission in response to their deteriorating condition. (c) Subsequent follow-up chest CT scans and EELV tests were conducted. Exclusion criteria encompassed: (a) Patients experiencing severe hemodynamic instability, defined as persistent systolic blood pressure below 90 mmHg, tachycardia exceeding 120 beats per minute, or bradycardia with evidence of poor cardiac output confirmed by point-of-care ultrasound examination. (b) Inability to complete the EELV test (e.g., due to a pronounced decline in SpO_2_ levels observed during the evaluation). (c) Recurrent ICU admissions. (d) Patients whose fraction of inspired oxygen (FiO_2_) exceeded 80%.

### Data collection

Patient demographics, date of disease onset, initial symptoms, duration of hospital admission and ICU admission, disease severity, comorbidities, chronic therapy, medications and treatment received during ICU, as well as chest CT scans, were extracted from electronic patient medical records. The EELV test was conducted within 48 h post-intubation, with follow-up assessments performed every 3–4 days for critically ill patients. The CT scans selected for analysis were obtained within a 24-hour window before or after the EELV measurements. EELV monitoring was discontinued for patients who underwent extubation, were discharged from ICU, or were no longer able to undergo further EELV measurements.

**Fig. 1:**
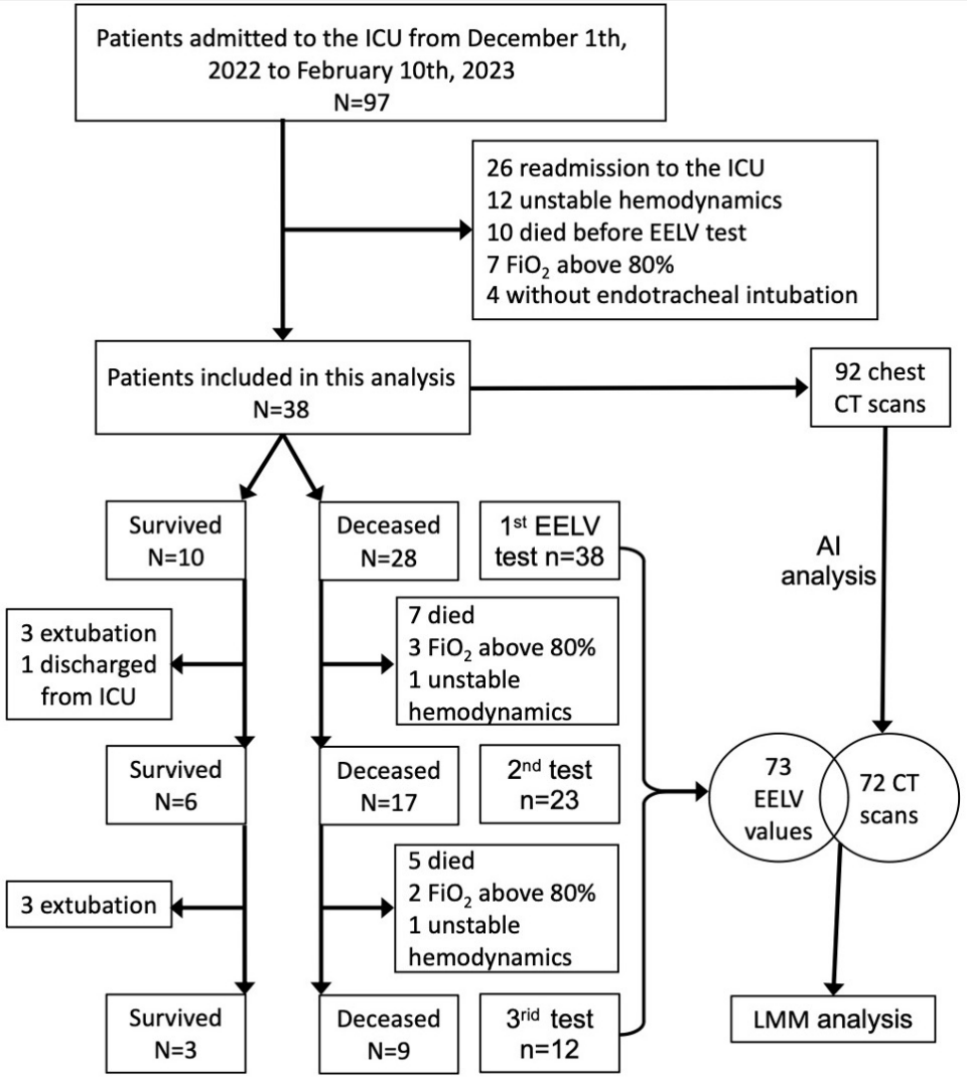
Participant flow diagram. LMM, Linear mixed model

This study involved EELV monitoring of a cohort of 38 patients: all 38 patients received an initial EELV assessment, 23 underwent a second evaluation, and 12 participated in a third round of monitoring. This resulted in a total of 73 EELV measurements across these patients. Additionally, 92 CT scans were performed, adhering to the specified temporal criteria for the study. Subsequent analysis using AI software enabled the successful identification and processing of 72 CT scans. However, the scans from 4 patients were not amenable to AI-based analysis. As a result, the final dataset for correlation analysis included 72 CT scans and their corresponding EELV values, encompassing a subset of 34 patients. The study flow is illustrated in Fig. 1.

### Ventilator parameters setting

The ventilator settings were aligned with established guidelines: patients were ventilated under A/C-VC or V-SIMV modes after intubation, initial tidal volume (VT) was set at 6–8 ml/kg of ideal body weight. Individualized positive end-expiratory pressure (PEEP) was titrated using the EIT-Costa method (Pulmo Vista 500, Dräger Medical) to maintain driving pressure below 15 cmH_2_O [17]. Key parameters such as peak pressure, plateau pressure (Pplat), respiratory rate (RR), minute ventilation, arterial pH, partial pressure of carbon dioxide (PaCO_2_), SpO_2_, and partial pressure of oxygen in arterial blood (PaO_2_) were continuously monitored. If Pplat exceeded 30 cmH_2_O or driving pressure exceeded 15 cmH_2_O, VT was gradually reduced to 4 ml/kg. Following VT reduction, RR was adjusted to ensure adequate minute ventilation. FiO_2_ was adjusted based on SpO_2_ and PaO_2_ levels. All patients received sedation (propofol, midazolam, dexmedetomidine) and analgesia (fentanyl) to achieve a target sedation level (RASS − 2 to 0) throughout their ICU stay. Rocuronium bromide was only used during FRC measurements to ensure accurate FRC calculations.

### EELV assessment

EELV was measured utilizing the nitrogen washout-washin technique (E-sCOVX module sensor, GE Healthcare, Madison, WI, USA). The infusion of intravenous anesthetic agents and rocuronium bromide was administered to establish controlled mechanical ventilation during EELV measurement. Consistency in ventilator parameters was maintained throughout the EELV monitoring including follow-up measurements. Other key ventilatory parameters, including PEEP, VT, RR, and static compliance of the respiratory system (Cstat), were also recorded from the mechanical ventilator at each measurement.

Lung Strain was calculated as:

$$Strain{\rm{ }} = {\rm{ }}VT/EELV$$

Predicted body weight (PBW) in kilograms (kg) was determined based on patient height measurements. These measurements were taken while the patient was in a supine position, using the following formula:

$$PBW{\rm{ }}\left( {male} \right){\rm{ }} = {\rm{ }}50{\rm{ }} + {\rm{ }}0.91{\rm{ }}\left( {heigh{t_{cm}} - {\rm{ }}152.4} \right)$$

$$PBW{\rm{ }}\left( {female} \right){\rm{ }} = {\rm{ }}45.5{\rm{ }} + {\rm{ }}0.91{\rm{ }}\left( {heigh{t_{cm}} - {\rm{ }}152.4} \right)$$

$$preFRC*{\rm{ }}\left( {male} \right){\rm{ }} = {\rm{ }}2.34{\rm{ }}heigh{t_{cm}} + {\rm{ }}0.01{\rm{ }}ag{e_{year}} - {\rm{ }}1.09{\rm{ }} \pm {\rm{ }}0.99$$

$$preFRC*{\rm{ }}\left( {female} \right){\rm{ }} = {\rm{ }}2.24{\rm{ }}heigh{t_{cm}} + {\rm{ }}0.001{\rm{ }}ag{e_{year}} - {\rm{ }}1.00{\rm{ }} \pm {\rm{ }}0.82$$

$$*{\rm{ }}preFRC:{\rm{ }}predicted\begin{array}{*{20}{c}}{}\end{array}functional\begin{array}{*{20}{c}}{}\end{array}residual\begin{array}{*{20}{c}}{}\end{array}capacity.$$

### CT image acquisition and volume analysis

A chest CT scan was performed based on clinical judgment, necessitated by changes in patient condition or for follow-up examination purposes. The scans were acquired using a 64-slice scanner (uCT 530+, R001; United Imaging, Shanghai, China) with patients in the supine position, under mechanical ventilation, covering the area from the lung bases to the apex. All CT acquisitions were performed without the use of contrast medium, adhering to the following parameters: tube voltage, 120 kVp; automatic exposure control for tube current; pitch, 0.5. Images were reconstructed with 0.5 mm slice thickness using sharp kernels and standard lung window settings (width, 1000 HU; level, -600 HU).

For the analysis of these chest CT scans, Dr. Pecker Diagnosis Robot (Pneumonia CT Image-Assisted Triage and Evaluation System V1.2) was employed. This is a sophisticated chest CT imaging analysis tool underpinned by deep learning technology. It uses a multi-task Unet network to segment the input chest CT images. Within the automatically segmented lung region and regions of interest/lesion regions, it calculates several metrics to quantify lung lesions: volumes and densities of the entire lung, individual left and right lungs, and separate lung lobes; lesion volumes, counts, densities, solid-to-total ratio, ground glass opacity ratio, as well as the ratios of bilateral lung ground glass opacity and consolidation volumes to the total lung volume. The implementation process and accuracy of this system have been validated in previously published studies [18].

### Statistical analysis

Linear mixed models (LMM), an extension of linear regression, offers a robust framework for analyzing correlated observations, such as repeated measures on the same subjects [19]. We employed LMM to assess differences in EELV, EELV/PBW and EELV/preFRC across survivor and non-survivor groups at each follow-up point (follow-up 1, 2 and 3). In this mixed model, patients were categorized as a random effect (random intercept), while time and group variables, along with their interaction term if significant, were treated as fixed effect. We also used LMM to examine the changes in EELV and their correlation with CT findings. Residual plots revealed no obvious deviations from homoscedasticity or normality. *P*-values, derived from likelihood-ratio tests that compare models with or without the specified effect, were considered statistically significant when *P* < 0.05. The agreement between preset and measured FRC gas volumes obtained through nitrogen washout/washin technique was evaluated with a Bland & Altman analysis. Continuous variables were presented as median [interquartile range], and categorical variables as frequency (%). All statistical analyses were performed using the R Project software, version 4.3.1, for macOS. Missing data were accounted for by using the mixed-effects model.

## Results

### General characteristics

During the study period, 38 of the 97 critically ill COVID-19 patients admitted to the ICU were included in the study (Fig. 1). Among them, 28 (73.7%) succumbed in the ICU, while 10 (26.3%) survived and were subsequently discharged to the ward. The average age of the patients was 70 years, indicating a predominantly elderly demographic. Their average BMI was 25 kg/m^2^, classifying them as overweight. There were no significant differences in age, BMI, median time from symptom onset to hospital admission (11 days), or median ICU stay (11 days) between the survivor and non-survivor groups. However, the survivors had a significantly longer total hospital stay than the non-survivors. The APACHE II score tended to be higher in the non-survivor group, although not statistically significant. Notably, a higher fluid balance of 347 mL [508,664 mL] was observed in the non-survivor group compared to the survivor group. Other clinical characteristics, including initial symptoms, disease severity, comorbidities, chronic therapy, and treatments received in the ICU, showed no statistical differences between the groups (Table 1, Table S1).

**Table 1:** Demographic and clinical characteristics of patients with CARDS

	All (*N* = 38)	Survivors (*N* = 10)	Non-survivors (*N* = 28)	*P*-value
Age (years)	72 [67,82]	72 [70,84]	76 [65,82]	0.982
Sex (male), n (%)	24 (63.2)	6 (60.0)	18 (64.3)	1.000
Weight (kg)	70 [58,78]	67 [61,75]	70 [57,80]	0.847
Height (cm)	168 [160,173]	163 [158,170]	169 [164,173]	0.146
Body mass index (kg/m^2^)	25 [20, 21]	24 [20, 22]	25 [21, 23]	0.519
Length from symptom onset to hospital admission (days)	11 [7, 19]	14 [4, 22]	10 [7, 14]	0.282
Length of hospitalization (days)	19 [14, 24]	31 [28,43]	18 [14, 23]	0.012*
Length of ICU stay (days)	11 [7, 17]	14 [12, 20]	9 [7, 14]	0.371
APACHE II	14 [8, 19]	9 [7, 13]	16 [12, 19]	0.079
Charlson score	2 [1, 3]	2 [1, 3]	2 [1, 3]	0.282
Liquid balance (mL/day)	306 [423,573]	160 [298,401]	347 [508,664]	0.025*
***Drugs received during ICU stay***				
Paxlovid, n (%)	19 (36.5)	2 (20.0)	17 (60.7)	0.065
Days of using Paxlovid	5 [3, 5]	5 [4, 5]	5 [2, 5]	0.709
Tocilizumab, n (%)	8 (21.1)	1 (10.0)	7 (25.0)	0.653
Methylprednisolone, n (%)	31 (81.6)	7 (70.0)	24 (85.7)	0.351
Days of using methylprednisolone	8 [5, 12]	6 [0,8]	10 [6, 13]	0.346
Heparin for prevention, n (%)	7 (18.4)	2 (20.0)	5 (17.9)	1.000
Heparin for treatment, n (%)	28 (73.7)	6 (60.0)	22 (78.6)	0.404
Fondaparinux Sodium, n (%)	4 (10.5)	0 (0.0)	4 (14.3)	0.556
Thymosin, n (%)	19 (50.0)	4 (40.0)	15 (53.6)	0.713
HIG, n (%)	7 (18.4)	4 (40.0)	3 (10.7)	0.063
Days of using HIG	5 [3, 6]	3 [3]	5 [4, 7]	0.567
CRRT, n (%)	16 (47.1)	4 (40.0)	12 (50.0)	0.715
***Bacteria***				
Acinetobacter baumannii (%)	33 (86.8)	9 (90.0)	24 (85.7)	1.000
Klebsiella pneumoniae (%)	18 (47.4)	5 (50.0)	13 (46.4)	1.000
Pseudomonas aeruginosa (%)	2 (5.26)	2 (20.0)	0 (0.0)	0.064
Stenotrophomonas maltophilia (%)	8 (21.1)	3 (30.0)	5 (17.9)	0.411
Staphylococcus aureus (%)	6 (15.8)	3 (30.0)	3 (10.7)	0.310

Values are [interquartile range] or number (%). CARDS: COVID-19 associated acute respiratory distress syndrome; ICU: intensive care unit; HIG: Human Immunoglobulin; CRRT: Continuous Renal Replacement Therapy. *P < 0.05

### EELV assessment

**Fig. 2:**
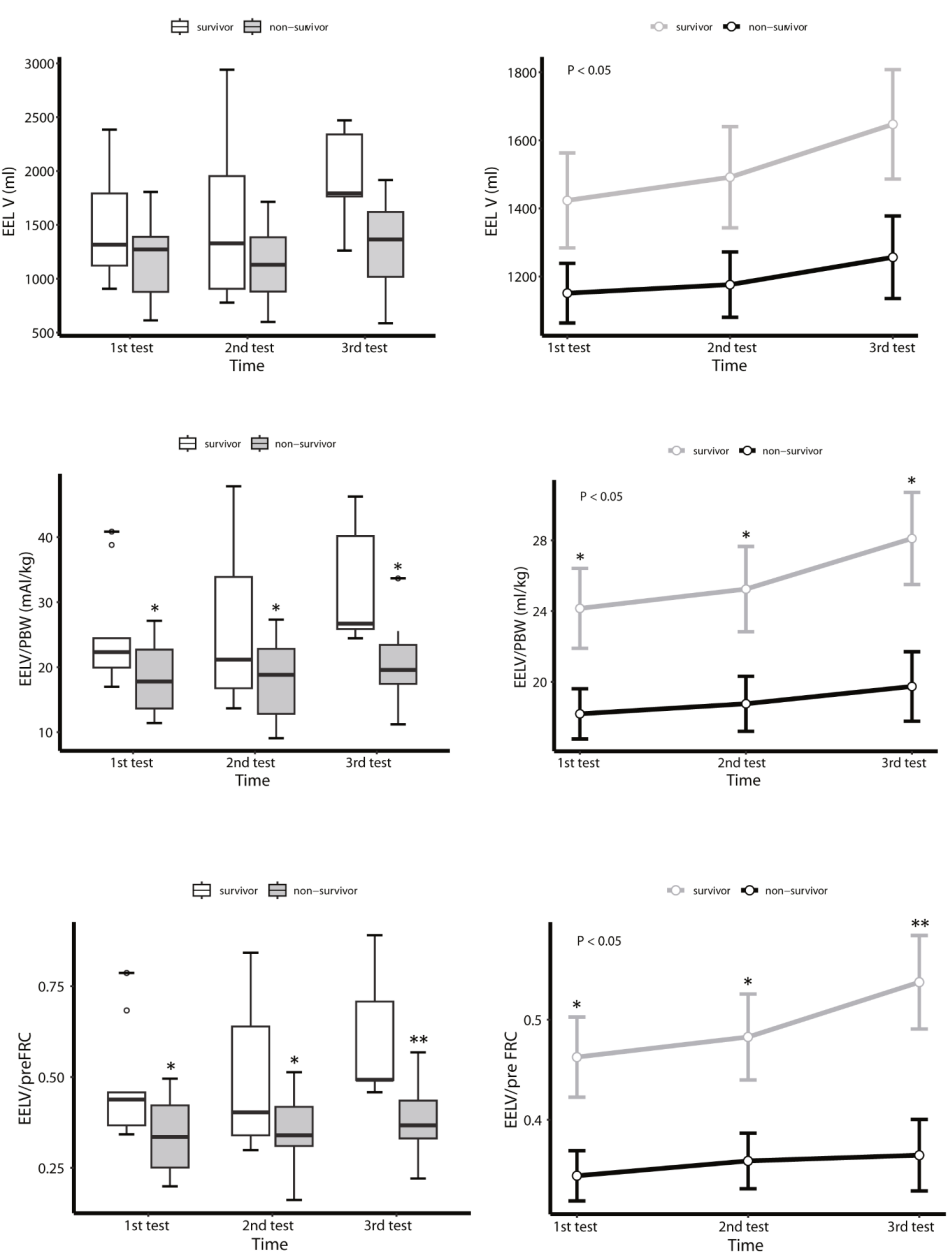
Changes in mean EELV (**A**), EELV/PBW (**B**), and EELV/preFRC (**C**) analyzed by LMM across three EELV tests. The horizontal line in the middle of each box (left column) indicates the median, the top and bottom borders of the box mark the 75th and 25th percentiles, the whiskers above and below the box indicate the 90th and 10th percentiles, and the points beyond the whiskers are outliers beyond the 90th or 10th percentiles. The modeled data (right column) show the standard error of marginal mean for the predicted values using the random-effects model. *P*-values signify the group effect. Asterisks (* or **) indicate statistically significant differences between the survival and death groups at each measurement. *P<0.05, ** *P*<0.01. (Also see Table S2 for details)

**Fig. 3:**
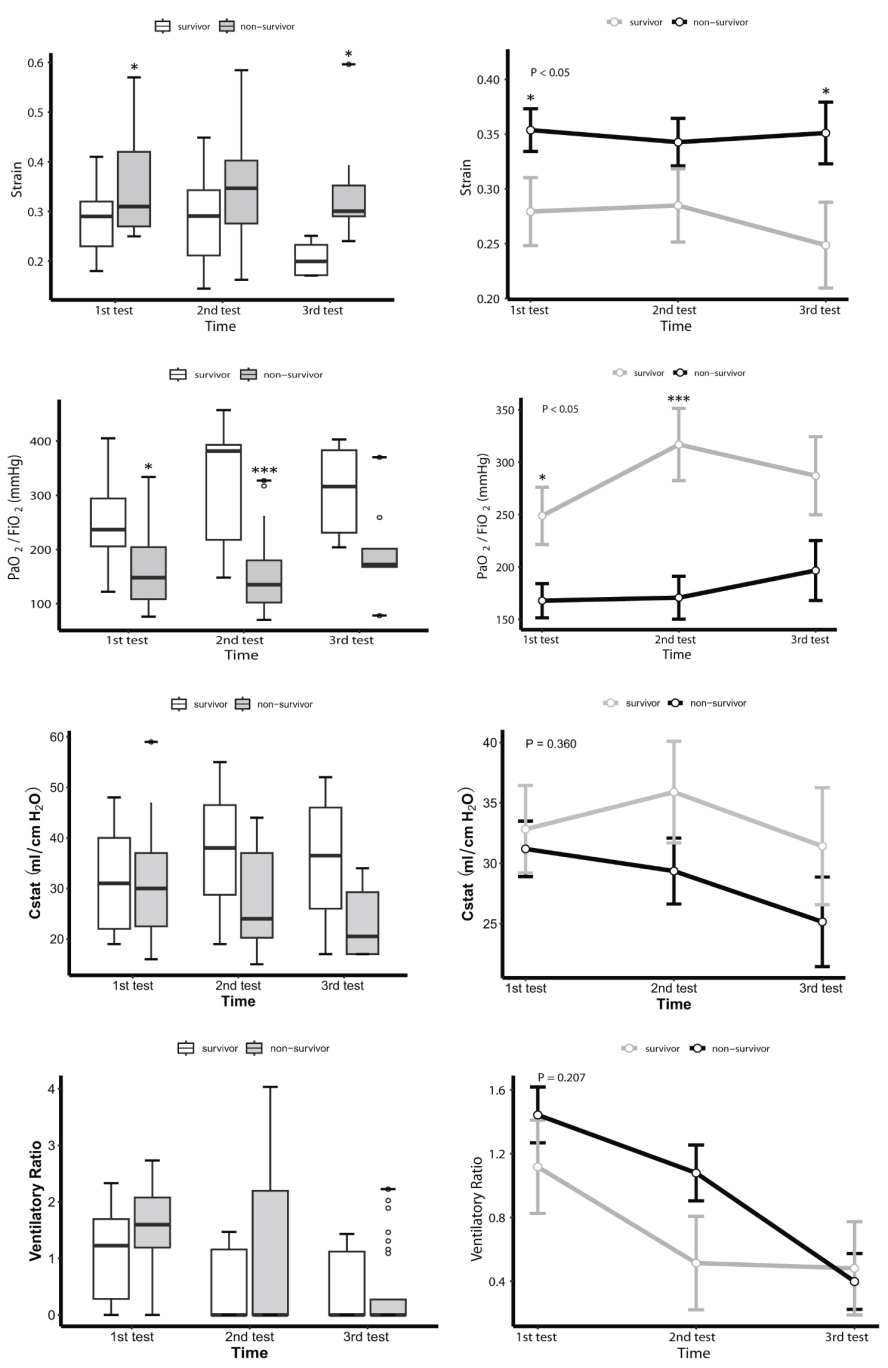
Changes in mean strain (**A**), PaO_2_/FiO_2_ (**B**), Cstat (**C**), and VR (**D**) analyzed by LMM across three EELV tests over 9–11 days. The horizontal line in the middle of each box (left column) indicates the median, the top and bottom borders of the box mark the 75th and 25th percentiles, the whiskers above and below the box indicate the 90th and 10th percentiles, and the points beyond the whiskers are outliers beyond the 90th or 10th percentiles. The modeled data (right column) show the standard error of marginal mean for the predicted values using the random-effects model. *P*-values signify the group effect. Asterisks (* or ***) indicate statistically significant differences between the survival and death groups at each measurement. *P<0.05, *** *P*<0.001

The initial ventilator settings for measuring EELV and the patient’s standard FRC showed no significant differences between the survivor group and the non-survivor group, except for FiO_2_ (50% vs. 60%, *P* = 0.036) (Table 2). Subsequently, we employed a mixed-effects model to compare EELV, EELV/PBW, and EELV/preFRC at three different time points between the groups, and found that in the survivor group, EELV (survivor vs. non-survivor group: *P*-group < 0.05, *P*-time = 0.065, *P*-group × time = 0.418), EELV/PBW (*P*-group < 0.05, *P*-time = 0.057, *P*-group × time = 0.341), and EELV/PreFRC (*P*-group < 0.05, *P*-time = 0.072, *P*-group × time = 0.289) were all significantly higher than in the non-survivor group (Table S2). While there were no significant variations in EELV/PBW and EELV/preFRC across the three follow-up sessions within the survivor group, a positive trend in EELV-related data over time was noted (Fig. 2, Table S2, and Table S5). Additionally, we compared the changes in strain, PaO_2_/FiO_2_ ratio, and Cstat between the groups across the three follow-up sessions. Strain was significantly lower in the survivor group (0.25 vs. 0.31, *P* = 0.032), with notable differences in the first and third sessions but not in the second. Nevertheless, no significant temporal changes in strain were observed within either group. Differences in the overall PaO_2_/FiO_2_ ratio were also noted between the survivor and the non-survivor group (168.5 vs. 248 mmHg, *P* = 0.001), with disparities in the first two follow-up sessions but not in the third. No differences in Cstat were observed between the groups (Fig. 3, Table S3 and Table S5).

**Table 2:** The ventilation parameters and preFRC values at baseline: analysis between survivor and non-survivor groups

	Total	Survival	Deceased	*P*-value
preFRC (ml)	3502.60 (2756.25, 3674.75)	3384.50 (2634.65, 3534.50)	3529.50 (2801.40, 3700.50)	0.182
VT (ml)	375.00 (350.00, 418.75)	375.00 (331.25, 418.75)	375.00 (350.00, 406.25)	0.48
Frequency (min)	19.00 (16.00, 25.00)	20.50 (15.25, 25.00)	19.00 (16.00, 25.00)	0.973
PEEP (cmH_2_O)	8.00 (6.00, 10.00)	8.00 (6.00, 8.00)	8.00 (6.00, 10.00)	0.564
FiO_2_ (%)	60.00 (50.00, 65.00)	50.00 (50.00, 50.00)	60.00 (50.00, 65.00)	0.036*
Cstat (ml/cmH_2_O)	22 (31,39)	31 (22,40)	30.5 (22.8,38.2)	0.952

Values are median (interquartile range). preFRC: predicted functional residual capacity; VT: tidal volume; PEEP: positive end-expiratory pressure; FiO_2_: fraction of inspired oxygen; Cstat: static compliance of the respiratory system. *P < 0.05

**Fig. 4:**
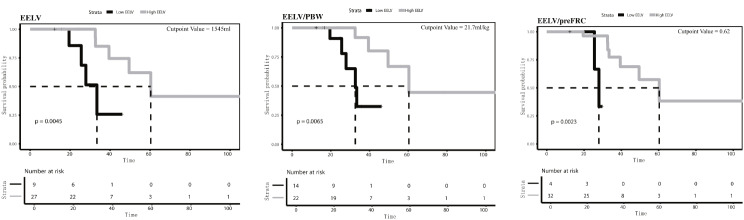
Determination of cutoff values for EELV (**A**), EELV/PBW (**B**), and EELV/pre FRC (**C**) using maximally selected Log-rank statistics. Based on these cutoff values, survival curves were plotted. The analysis focuses on patient mortality during ICU hospitalization, with ‘event’ signifying death and ‘time’ denoting the period from symptom onset to either death or discharge for surviving patients. The dashed line on the y-axis signifies a survival probability of 0.5, intersecting with the survival curve to indicate the estimated median survival time

To ascertain optimal cutoff values for EELV, EELV/PBW, and EELV/preFRC, we employed the Maximally Selected Log-rank Statistic for multiple classifications. Subsequently, we generated survival curves depicting patient outcomes from symptom onset to death. With a cutoff value of 1545 ml for EELV, the median survival time in the high EELV group was notably longer (60.3 days), compared to the low EELV group (27.9 days). This significant difference in survival times was confirmed by the Log-rank test (*P* < 0.05) (Fig. 4A). Similarly, with a cutoff value of 21.7 ml/kg for EELV/PBW, the median survival time was substantially greater in the high EELV/PBW group (115.4 days) than in the low EELV/PBW group (32.7 days), with the Log-rank test indicating a significant difference (*P* < 0.05) (Fig. 4B). Likewise, utilizing a cutoff value of 0.62 for EELV/preFRC, we observed that the median survival time in the high EELV/preFRC group (60.3 days) exceeded that in the low EELV/preFRC group (33.4 days). The Log-rank test exhibited a significant difference between the groups (*P* < 0.05) (Fig. 4C). These findings suggest that patients categorized in the high EELV, EELV/PBW, or EELV/preFRC groups not only have a greater likelihood of survival at a given time point, but also exhibit better overall survival outcomes.

Based on the findings of Sinha et al. (2019) [20], the ventilatory ratio (VR), calculated as *[minute ventilation (ml/min) × PaCO*_*2*_*(mm Hg)]/(predicted body weight × 100 × 37.5)*, plays a crucial role in predicting outcomes in ARDS. In the current study, analysis of ventilator ratio using LMM showed no significant difference between the survivor and non-survivor groups (*P*-group = 0.207, *P*-time < 0.05, *P*-group × time = 0.363) (Fig. 3, Table S3). Furthermore, it was observed that the ventilatory ratio in both the survivor and non-survivor groups decreased significantly over time.

### Comparison of EELV and AI-analyzed CT volumetry

**Fig. 5:**
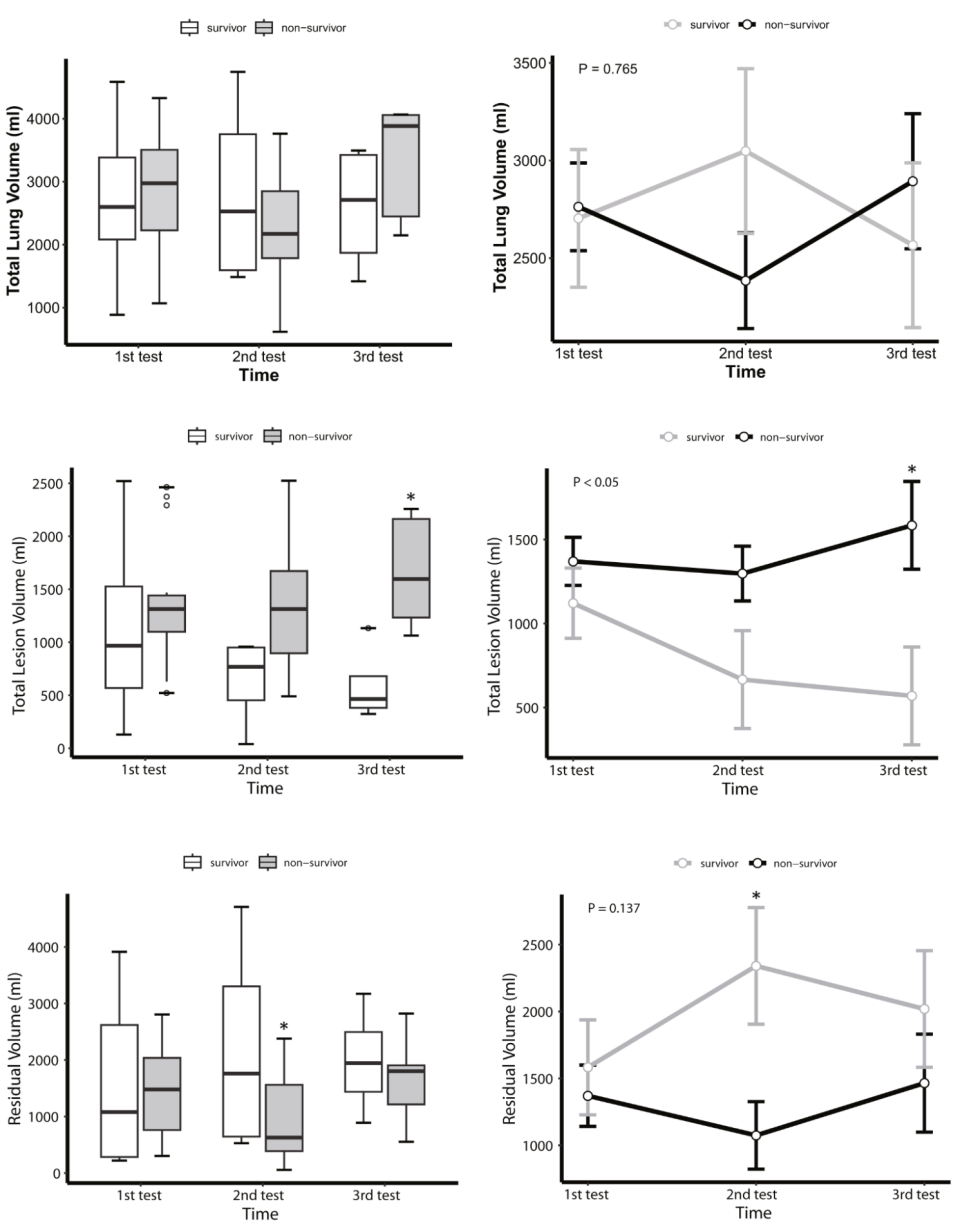
Changes in mean total lung volume (**A**), total lesion volume (**B**), and residual (**C**) analyzed by LMM across three EELV tests over 9–11 days. The horizontal line in the middle of each box (left column) indicates the median, the top and bottom borders of the box mark the 75th and 25th percentiles, the whiskers above and below the box indicate the 90th and 10th percentiles, and the points beyond the whiskers are outliers beyond the 90th or 10th percentiles. The modeled data (right column) show the standard error of marginal mean for the predicted values using the random-effects model. *P*-values signify the group effect. Asterisks (*) indicate statistically significant differences between the survival and death groups at each measurement. *P<0.05

**Fig. 6:**
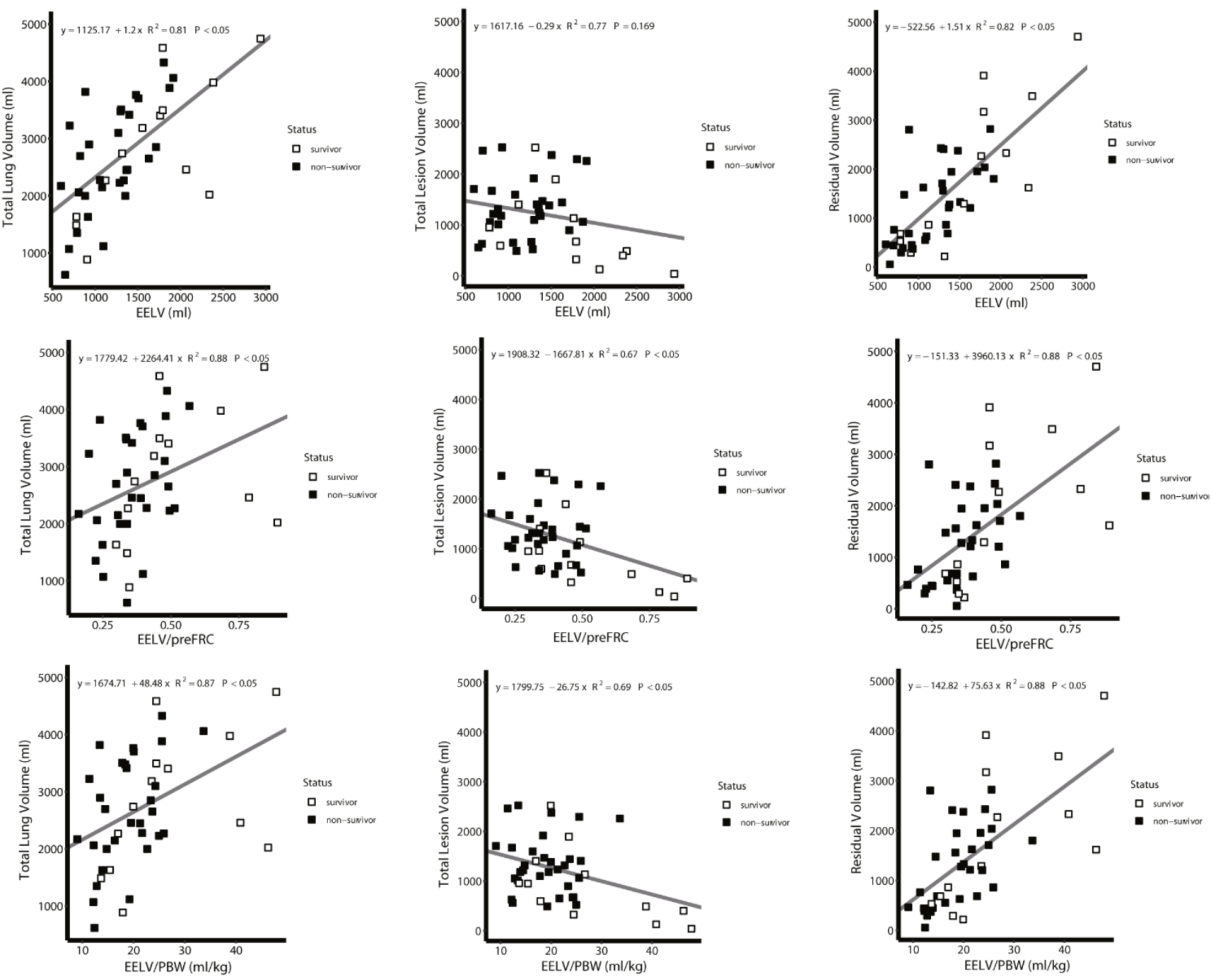
Correlations calculated using LMM between EELV measured by multiple breath nitrogen washout/washin technique versus computed tomography. The correlations of EELV (**A**), EELV/preFRC (**B**), and EELV/PBW (**C**) with total lung volume (left), total lesion volume (middle), and residual volume (right)

CT-graphic volumetry of total lung volume, lesion volume, and residual lung volume was performed using AI software. Comparisons were then drawn between the groups of survivors and non-survivors (Table S4). As illustrated in Fig. 5, no significant differences were observed in total lung volume and residual lung volume between the groups. However, the survivor group exhibited significantly lower total lesion volume than the non-survivor group (survivor vs. non-survivor group: *P*-group < 0.05, *P*-time = 0.348, *P*-group × time = 0.056) (Table S4). Further analysis using LMM method was conducted to explore the correlation between EELV-related parameters and total lung volume, lesion volume, and residual lung volume calculated by AI software. Figure 6A shows a positive correlation between EELV and both total lung volume (R^2^ = 0.81, *P* < 0.05) and residual lung volume (R^2^ = 0.82, *P* < 0.05), but no correlation with lesion volume. In Fig. 6B, a positive correlation was found between EELV/preFRC and total lung volume (R^2^ = 0.88, *P* < 0.05) and residual lung volume (R^2^ = 0.88, *P* < 0.05). Additionally, a negative correlation was noted with injured lung volume (R^2^ = 0.67, *P* < 0.05). Similarly, Fig. 6C demonstrates a positive correlation between EELV/PBW and total lung volume (R^2^ = 0.87, *P* < 0.05) and residual lung volume (R^2^ = 0.88, *P* < 0.05), and a negative correlation with injured lung volume (R^2^ = 0.69, *P* < 0.05). Furthermore, a notable discrepancy of 471.10 ml was identified between the residual lung volume as calculated by the AI software and the one measured by EELV.

## Discussion

We evaluated the values and changes of EELV in patients with CARDS and found certain association between EELV and their prognosis, as well as a significant correlation with AI-analyzed CT lung volumes. However, in both the survivor group and non-survivor group, solely observing changes in CT lesion volume or the PaO_2_/FiO_2_ ratio did not consistently yield differences at every measurement point. While there are some reports on pulmonary function changes post-discharge, literature regarding EELV and its variations in CARDS patients under invasive mechanical ventilation is limited. To our knowledge, this study is pioneering in demonstrating that EELV can be an effective indicator of lung damage extent in CARDS patients and provide valuable insights into their prognosis. Our analysis includes comparisons of EELV differences and trends in COVID-19 patients, potentially informing assessments and prognoses for patients with ARDS from other causes. Monitoring EELV could potentially serve as an alternative to repetitive CT scans for tracking lung lesion progression in patients with CARDS, offering a quicker and more convenient method for follow-up.

COVID-19 can progress to ARDS, necessitating mechanical ventilation in approximately one-third of critically ill patients [21]. Notably, during the initial wave of the pandemic, the mortality rates among patients receiving invasive mechanical ventilation varied widely, ranging from 23.3 to 81% [22, 23]. In our study, we investigated the ICU mortality rate of patients with CARDS and invasive mechanical ventilation after Shanghai’s first lockdown ended. The ICU mortality rate for these patients was 73.7%. Previous studies have indicated that ARDS typically develops around 8–9 days after the onset of COVID-19 symptoms. In our cohort, the average time from symptom onset to hospital admission was 11 days, with no significant difference between the survivor and non-survivor groups. This timeline could be attributed to the overwhelming surge of COVID-19 cases, which strained healthcare resources, leading to hospital bed shortages, personnel constraints, and limited availability of medications and equipment. Consistent with previous studies, factors like advanced age, comorbidities, and obesity were associated with poorer outcomes and prognosis in our patient groups. The average age of our patients was 72 years, and they generally exhibited an overweight status, with a mean BMI of 25 (kg/m^2^). Although both the survivor and non-survivor groups had a Charlson Comorbidity Index score of 2, we observed a higher proportion of non-survivors with comorbidities such as kidney disease, cardiovascular disorders, and pulmonary diseases. Additionally, the APACHE II score tended to be higher in the non-survivor group, though it did not reach statistical significance. It should be noted, however, that the limited sample size of our study may have influenced these findings.

Fluid management in patients with ARDS presents challenges and controversies. In ARDS patients, a positive fluid balance is associated with prolonged mechanical ventilation, extended stays in ICU and hospital, and higher mortality [24]. Research on patients with CARDS has also shown that a higher cumulative fluid balance is associated with a longer ventilation duration [25]. The current study noted that the non-survivors had higher fluid balances compared to the survivors, likely due to increased pulmonary endothelial and epithelial permeability in ARDS, leading to fluid leakage into pulmonary interstitium and alveoli space. Therefore, fluid management should prioritize adequate oxygen delivery while avoiding exacerbating pulmonary edema, which could impair gas exchange [26]. However, a recent study revealed a significant interaction between phenotypes and fluid management strategy on 60-day mortality. The main interpretation is that the fluid balance trajectories are dynamic, while the predictive value of static values is limited [27]. Both fluid perfusion and tissue edema can affect EELV, but further research is needed to fully understand the relationship between fluid balance and EELV, as well as the characteristics of EELV in different ARDS subtypes.

Low lung function is recognized as a strong and independent risk factor for all-cause mortality [28, 29]. However, previous studies have primarily focused on general populations or chronic disease cohorts, emphasizing FEV1 and FVC as the primary indicators [30]. Yet, there seems to be hesitancy in acknowledging lung function as an independent marker of disease severity. In patients discharged after severe or critical COVID-19, reduced respiratory function is a significant issue [31]. While blood gas analysis and CT scans are useful in assessing a patient’s oxygenation capacity and detecting structural changes in the lungs, they fall short of providing a comprehensive evaluation of lung function. In this study, we propose that measures associated with EELV offer a more direct assessment of residual lung function, with potential correlations to patient prognosis. Our findings reveal a significant decline in EELV among patients with CARDS receiving mechanical ventilation. Dilken et al. conducted a study on 40 intubated COVID-19 patients to examine the variations in EELV while in supine and prone positions. Their study monitored changes over a single day, and reported median values of 1444 ml for EELV, 23.4 ml/kg for EELV/PBW, and 0.31 for strain in the supine position, but did not assess patient outcomes [32]. In our study, we found median values of 1287 ml for EELV, 19.96 ml/kg for EELV/PBW, and 0.30 for strain. Notably, EELV, EELV/PBW, and EELV/preFRC were consistently lower in the non-survivor group compared to the survivor group. Furthermore, the established cutoff values for EELV, EELV/PBW, and EELV/preFRC effectively differentiated patients into two distinct groups with varying survival times and prognoses. These findings suggest that EELV and its associated parameters could be crucial in determining the prognostic outcomes of patients with CARDS.

The selection of PEEP levels during mechanical ventilation can impact the assessment of EELV. To address potential statistical challenges arising from reduced sample sizes in later stages, we maintained consistent mechanical ventilation parameter settings for each EELV measurement. Through prospective data collection at baseline and at regular intervals during treatment, we conducted statistical analyses encompassing EELV, EELV/PBW, EELV/preFRC, as well as indices such as strain, P/F ratio, Cstat, and ventilatory ratio.

Our study had a relatively small sample size; therefore, we utilized statistical analysis techniques including LMM and Maximally Selected Log-rank Statistic, which might have yielded reliable estimates even with limited sample sizes.

Our findings revealed significant differences in EELV between survivors and non-survivors. Using an EELV of 1545 ml as a cutoff value, we observed an extended median survival time in survivors with CARDS compared to non-survivors. In other words, patients with CARDS who had an EELV above this value had a longer median survival time compared to those with an EELV below 1545 ml.

In our study, although EELV and its associated parameters demonstrated a strong correlation with CT-measured lung volumes (including total lung volume and residual lung volume), CT measurements alone did not reveal significant differences between the survivor and non-survivor groups, except for lesion volume. Additionally, although the PaO_2_/FiO_2_ ratio showed an overall difference between the groups, this difference lost statistical significance during the third follow-up measurement. Interestingly, both EELV/PBW and EELV/preFRC exhibited statistically significant differences between the survivor and non-survivor groups, both in the overall analysis and across the three measurement points. Lieuwe Bos et al. reported that while the PaO_2_/FiO_2_ ratio is an important prognostic indicator for patients with CARDS, the related mechanical ventilation parameters such as mechanical power and ventilatory ratio hold greater significance in guiding patient prognosis and classification over time [33]. Consistent with these observations, our study also noted that while PaO_2_/FiO_2_ ratio did not vary significantly between the survivor and non-survivor groups over time, a growing disparity was evident in EELV-related indicators. These findings suggest that EELV measurement may offer a more effective tool for evaluation and follow-up compared to PaO_2_/FiO_2_ and CT scans for assessing lung function and prognosis in CARDS patients. However, further studies are required to validate these results and understand their clinical implications.

This study has some limitations. Firstly, as a single center study, its findings necessitate further validation through broader research. Secondly, although data collection was prospective, the study’s retrospective nature may impact the generalizability of the conclusions. The study also had a relatively small sample size. Moreover, CT and EELV measurements were not conducted in real-time but rather within a 24-hour window surrounding each intervention. This approach may not accurately reflect the rapid and dynamic changes in patient conditions. Finally, while CT scans are the gold standard for assessing functional residual capacity, in this study, patients underwent CT imaging using a transport ventilator, which raises concerns about the consistency of capturing scans at end expiration, and could potentially affect lung volume evaluations.

## Conclusions

In summary, this study represents a pioneering exploration of the changes in EELV among surviving and deceased patients with CARDS. Our findings reveal significant differences in EELV between surviving and deceased patients and establish a strong correlation between EELV and CT evaluations of lung volume. These insights contribute to our understanding of the progression of pulmonary lesions in critically ill COVID-19 patients, particularly during the follow-up of endotracheal intubation. In addition to traditional assessments like CT evaluations and the PaO_2_/FiO_2_ ratio, the monitoring of EELV and related indicators may offer a novel approach for evaluating the condition and prognosis of patients with ARDS caused by other factors.

### Electronic supplementary material

Below is the link to the electronic supplementary material.

Supplementary Material 1

## Data Availability

Data are available on request from the corresponding author.

## Abbreviations

ARDS: Acute respiratory distress syndrome
CARDS: COVID-19 associated acute respiratory distress syndrome
Cstat: Static compliance of the respiratory system
CT: Computed tomography
EELV: End-expiratory lung volume
FRC: Functional residual capacity
LMM: Linear mixed models
PaO_2_/FiO_2_: Arterial oxygen partial pressure to the fraction of inspired oxygen
PBW: Predicted body weight
PEEP: Positive end-expiratory pressure
Pplat: Plateau pressure
preFRC: Predicted functional residual capacity
RR: Respiratory rate
SpO_2_: Oxygen saturation
VR: Ventilatory ratio
VT: Tidal volume

## References

[1] Attaway AH, Scheraga RG, Bhimraj A, Biehl M, Hatipoğlu U (2021). Severe covid-19 pneumonia: pathogenesis and clinical management. BMJ.

[2] Hatabu H, Kaye KM, Christiani DC (2023). Viral infection, Pulmonary Fibrosis, and Long COVID. Am J Respir Crit Care Med.

[3] Shaw RJ, Bradbury C, Abrams ST, Wang G, Toh CH (2021). COVID-19 and immunothrombosis: emerging understanding and clinical management. Br J Haematol.

[4] Gattinoni L, Carlesso E, Cressoni M (2011). Assessing gas exchange in acute lung injury/acute respiratory distress syndrome: diagnostic techniques and prognostic relevance. Curr Opin Crit Care.

[5] Barbas CS, Isola AM, Caser EB (2014). What is the future of acute respiratory distress syndrome after the Berlin definition?. Curr Opin Crit Care.

[6] Matthay MA, Arabi Y, Arroliga AC, Bernard G, Bersten AD, Brochard LJ, Calfee CS, Combes A, Daniel BM, Ferguson ND (2024). A New Global Definition of Acute Respiratory Distress Syndrome. Am J Respir Crit Care Med.

[7] Ranieri VM, Rubenfeld G, Slutsky AS (2023). Rethinking Acute Respiratory Distress Syndrome after COVID-19: if a better definition is the answer, what is the question?. Am J Respir Crit Care Med.

[8] van der Wal LI, Grim CCA, Del Prado MR, van Westerloo DJ, Boerma EC, Rijnhart-de Jong HG, Reidinga AC, Loef BG, van der Heiden PLJ, Sigtermans MJ et al. Conservative versus liberal oxygenation targets in Intensive Care Unit patients (ICONIC): a Randomized Clinical Trial. Am J Respir Crit Care Med 2023. 10.1164/rccm.202303-0560OC PMC10563190 37552556

[9] Barrot L, Asfar P, Mauny F, Winiszewski H, Montini F, Badie J, Quenot JP, Pili-Floury S, Bouhemad B, Louis G (2020). Liberal or conservative oxygen therapy for Acute Respiratory Distress Syndrome. N Engl J Med.

[10] Gommers D (2014). Functional residual capacity and absolute lung volume. Curr Opin Crit Care.

[11] Leith DE, Brown R (1999). Human lung volumes and the mechanisms that set them. Eur Respir J.

[12] Berger-Estilita J, Haenggi M, Ott D, Berger D (2021). Accuracy of the end-expiratory lung volume measured by the modified nitrogen washout/washin technique: a bench study. J Transl Med.

[13] Dellamonica J, Lerolle N, Sargentini C, Beduneau G, Di Marco F, Mercat A, Richard JC, Diehl JL, Mancebo J, Rouby JJ (2011). Accuracy and precision of end-expiratory lung-volume measurements by automated nitrogen washout/washin technique in patients with acute respiratory distress syndrome. Crit Care.

[14] Luecke T, Meinhardt JP, Herrmann P, Klemm S, Weiss A, Weisser G, Hirschl RB, Quintel M (2003). End-expiratory lung volumes and density distribution patterns during partial liquid ventilation in healthy and oleic acid-injured sheep: a computed tomography study. Crit Care Med.

[15] Graf J, Santos A, Dries D, Adams AB, Marini JJ (2010). Agreement between functional residual capacity estimated via automated gas dilution versus via computed tomography in a pleural effusion model. Respir Care.

[16] Patel A, Jernigan DB (2020). Initial Public Health Response and Interim Clinical Guidance for the 2019 Novel Coronavirus Outbreak - United States, December 31, 2019-February 4, 2020. MMWR Morb Mortal Wkly Rep.

[17] Sella N, Pettenuzzo T, Zarantonello F, Andreatta G, De Cassai A, Schiavolin C, Simoni C, Pasin L, Boscolo A, Navalesi P (2021). Electrical impedance tomography: a compass for the safe route to optimal PEEP. Respir Med.

[18] Shi H, Xu Z, Cheng G, Ji H, He L, Zhu J, Hu H, Xie Z, Ao W, Wang J (2022). CT-based radiomic nomogram for predicting the severity of patients with COVID-19. Eur J Med Res.

[19] Liu S, Rovine MJ, Molenaar PC (2012). Selecting a linear mixed model for longitudinal data: repeated measures analysis of variance, covariance pattern model, and growth curve approaches. Psychol Methods.

[20] Armstrong RA, Kane AD, Cook TM (2020). Outcomes from intensive care in patients with COVID-19: a systematic review and meta-analysis of observational studies. Anaesthesia.

[21] Wu F, Shi S, Wang Z, Wang Y, Xia L, Feng Q, Hang X, Zhu M, Zhuang J (2024). Identifying novel clinical phenotypes of acute respiratory distress syndrome using trajectories of daily fluid balance: a secondary analysis of randomized controlled trials. Eur J Med Res.

[22] Burney PG, Hooper R (2011). Forced vital capacity, airway obstruction and survival in a general population sample from the USA. Thorax.

[23] Zhou F, Yu T, Du R, Fan G, Liu Y, Liu Z, Xiang J, Wang Y, Song B, Gu X (2020). Clinical course and risk factors for mortality of adult inpatients with COVID-19 in Wuhan, China: a retrospective cohort study. Lancet.

[24] Reyna ME, Bedard MA, Subbarao P (2023). Lung function as a biomarker of Health: An Old Concept Rediscovered. Am J Respir Crit Care Med.

[25] Sinha P, Calfee CS, Beitler JR, Soni N, Ho K, Matthay MA, Kallet RH (2019). Physiologic analysis and clinical performance of the ventilatory ratio in Acute Respiratory Distress Syndrome. Am J Respir Crit Care Med.

[26] Krause M, Douin DJ, Kim KK, Fernandez-Bustamante A, Bartels K (2021). Characteristics and outcomes of mechanically ventilated COVID-19 Patients-An Observational Cohort Study. J Intensive Care Med.

[27] Giovanni SP, Seitz KP, Hough CL (2024). Fluid Management in Acute Respiratory failure. Crit Care Clin.

[28] Ahuja S, de Grooth HJ, Paulus F, van der Ven FL, Serpa Neto A, Schultz MJ, Tuinman PR (2022). Association between early cumulative fluid balance and successful liberation from invasive ventilation in COVID-19 ARDS patients - insights from the PRoVENT-COVID study: a national, multicenter, observational cohort analysis. Crit Care.

[29] Vignon P, Evrard B, Asfar P, Busana M, Calfee CS, Coppola S, Demiselle J, Geri G, Jozwiak M, Martin GS (2020). Fluid administration and monitoring in ARDS: which management?. Intensive Care Med.

[30] Leivseth L, Nilsen TI, Mai XM, Johnsen R, Langhammer A (2014). Lung function and respiratory symptoms in association with mortality: the HUNT study. Copd.

[31] Bellan M, Soddu D, Balbo PE, Baricich A, Zeppegno P, Avanzi GC, Baldon G, Bartolomei G, Battaglia M, Battistini S (2021). Respiratory and psychophysical sequelae among patients with COVID-19 four months after Hospital Discharge. JAMA Netw Open.

[32] Dilken O, Rezoagli E, Yartaş Dumanlı G, Ürkmez S, Demirkıran O, Dikmen Y (2022). Effect of prone positioning on end-expiratory lung volume, strain and oxygenation change over time in COVID-19 acute respiratory distress syndrome: a prospective physiological study. Front Med (Lausanne).

[33] Bos LDJ, Sjoding M, Sinha P, Bhavani SV, Lyons PG, Bewley AF, Botta M, Tsonas AM, Serpa Neto A, Schultz MJ (2021). Longitudinal respiratory subphenotypes in patients with COVID-19-related acute respiratory distress syndrome: results from three observational cohorts. Lancet Respir Med.

