# Supplementary material for: End-expiratory lung volumes as a potential indicator for COVID-19 associated acute respiratory distress syndrome: a retrospective study
Source: BMC Pulm Med. 2024 Jun 25;24:298. doi: 10.1186/s12890-024-03118-2 (PMC11197326; doi:10.1186/s12890-024-03118-2)
Supplement: Supplementary file 1 — Supplementary Material 1 [file 12890_2024_3118_MOESM1_ESM.docx]

**Table S1.** Other demographic and clinical characteristics of patients with CARDS

|  | | All (N=38) | Survivors (N=10) | Non-survivors (N=28) | | *P-*value |
| --- | --- | --- | --- | --- | --- | --- |
| ***Initial symptoms*** |  | |  | |  |  |
| Chest tightness, n (%) | 10 (19.2) | | 3 (30.0) | | 7 (25.0) | 1.000 |
| Cough, n (%) | 15 (28.8) | | 3 (30.0) | | 12 (42.9) | 0.709 |
| Fever, n (%) | 25 (48.1) | | 8 (80.0) | | 17 (60.7) | 0.441 |
| Sputum, n (%) | 7 (13.5) | | 0 (0.0) | | 7 (25.0) | 0.156 |
| **Chronic therapy** |  | |  | |  |  |
| Calcium channel blockers, n (%) | 17 (44.7) | | 6 (60.0) | | 11 (39.3) | 0.293 |
| Beta blocker, n (%) | 11 (28.9) | | 1 (10.0) | | 10 (35.7) | 0.225 |
| ACEI/ARD, n (%) | 9 (23.7) | | 3 (30.0) | | 6 (21.4) | 0.673 |
| Oral anticoagulants, n (%) | 3 (7.9) | | 1 (10.0) | | 2 (7.1) | 1.000 |
| Antiplatelet therapy, n (%) | 7 (18.4) | | 2 (20.0) | | 5 (17.9) | 1.000 |
| Steroids in the previous month, n (%) | 6 (15.8) | | 0 (0.0) | | 6 (21.4) | 0.168 |
| Lipid-lowering medication, n (%) | 8 (21.1) | | 2 (20.0) | | 6 (21.4) | 1.000 |
| antidiabetic medication, n (%) | 8 (21.1) | | 3 (30.0) | | 5 (17.9) | 0.411 |
| Immunosuppressant drug, n (%) | 6 (15.8) | | 1 (10.0) | | 5 (17.9) | 1.000 |
| **Comorbidities** |  | |  | |  |  |
| CTD | 6 (15.8) | | 2 (20.0) | | 4 (14.3) | 0.644 |
| Tumor, n (%) | 12 (31.6) | | 4 (40.0) | | 8 (28.6) | 0.694 |
| Cardiovascular disease |  | |  | |  |  |
| Arrhythmia, n (%) | 5 (13.2) | | 2 (20.0) | | 3 (10.7) | 0.592 |
| Hypertension, n (%) | 26 (68.4) | | 7 (70.0) | | 19 (67.9) | 1.000 |
| CAD, n (%) | 11 (28.9) | | 2 (20.0) | | 9 (32.1) | 0.690 |
| Cerebrovascular disease |  | |  | |  |  |
| PD/Dementia/Brain atrophy, n (%) | 4 (10.5) | | 1 (10.0) | | 3 (10.7) | 1.000 |
| ICH, n (%) | 2 (5.3) | | 1 (10.0) | | 1 (3.6) | 0.462 |
| Brain infarction, n (%) | 1 (2.6) | | 0 (0.0) | | 1 (3.6) | 1.000 |
| Pulmonary diseases |  | |  | |  |  |
| COPD, n (%) | 3 (7.9) | | 1 (10.0) | | 2 (7.1) | 1.000 |
| Bronchiectasis, n (%) | 3 (7.9) | | 1 (10.0) | | 2 (7.1) | 1.000 |
| ILD, n (%) | 3 (7.9) | | 0 (0.0) | | 3 (10.7) | 0.552 |
| TB, n (%) | 2 (5.3) | | 0 (0.0) | | 2 (7.1) | 1.000 |
| Others, n (%) | 1 (2.6) | | 0 (0.0) | | 1 (3.6) | 1.000 |
| Endocrine diseases |  | |  | |  |  |
| DM, n (%) | 11 (28.9) | | 3 (30.0) | | 8 (28.6) | 1.000 |
| Hyperlipidemia, n (%) | 1 (2.6) | | 0 (0.0) | | 1 (3.6) | 1.000 |
| Others, n (%) | 3 (7.9) | | 1 (10.0) | | 2 (7.1) | 1.000 |
| Kidney diseases |  | |  | |  |  |
| CKD, n (%) | 8 (21.1) | | 0 (0.0) | | 8 (28.6) | 0.082 |
| CKD（stage） | 3 (2,5) | | 0 (0,0) | | 3 (2,5) | 0.063 |
| HD, n (%) | 2 (5.3) | | 0 (0.0) | | 2 (7.1) | 1.000 |
| Renal Transplantation, n (%) | 2 (5.3) | | 0 (0.0) | | 2 (7.1) | 1.000 |
| Digestive diseases |  | |  | |  |  |
| HBV/Liver cirrhosis, n (%) | 4 (10.5) | | 0 (0,0) | | 4 (14.3) | 0.556 |
| Gastritis/GU/Gastrorrhagia, n (%) | 5 (13.2) | | 2 (20.0) | | 3 (10.7) | 0.592 |
| Others, n (%) | 11 (28.9) | | 1 (10.0) | | 10 (35.7) | 0.225 |

Values are median (interquartile range) or number (%). CTD: connective tissue disease; CAD: coronary artery disease; PD: Parkinson’s disease; ICH: intracerebral hemorrhage; COPD: chronic obstructive pulmonary disease; ILD: interstitial lung disease; TB: tuberculosis; DM: Diabetes Mellitus; CKD: chronic kidney disease; HD: hemodialysis; HBV: Hepatitis B Virus infection; GU: Gastric Ulcer.

**Table S2.** The EELV, EELV/ PBW and EELV/preFRC between the groups of survivors and non-survivors at each follow-up assessment time point (1, 2 and 3) analyzed by LMM.

| Variable | Coefficient | Estimate | Std. Error | Statistic Value | *P*-value |
| --- | --- | --- | --- | --- | --- |
| **EELV** |  |  |  |  |  |
| *Main Effect* | Group | 303.03 | 154.18 | 3.84 | 0.049^*^ |
|  | Time | 69.19 | 37.64 | 3.39 | 0.065 |
|  | Time (Non-survivors) | 46.16 | 41.28 | 1.28 | 0.258 |
|  | Time (Survivors) | 104.42 | 76.79 | 1.93 | 0.164 |
| *Conditional Effect* | Non-survivor - Survivor (Time = 1) | -273 | 165 | -1.66 | 0.105 |
|  | Non-survivor - Survivor (Time = 2) | -316 | 177 | -1.78 | 0.081 |
|  | Non-survivor - Survivor (Time = 3) | -391 | 201 | -1.94 | 0.057 |
|  | Time 1 - Time 2 (Non-survivors) | -25.2 | 76.7 | -0.33 | 0.942 |
|  | Time 1 - Time 3 (Non-survivors) | -105.6 | 106.6 | -0.99 | 0.588 |
|  | Time 2 - Time 3 (Non-survivors) | -80.4 | 107.5 | -0.75 | 0.737 |
|  | Time 1 - Time 2 (Survivors) | -68.1 | 118.8 | -0.57 | 0.835 |
|  | Time 1 - Time 3 (Survivors) | -223.6 | 128.9 | -1.73 | 0.209 |
|  | Time 2 - Time 3 (Survivors) | -155.5 | 130.0 | -1.20 | 0.465 |
| *Interaction Effect* | *Group × Time* | 61.78 | 78.40 | 0.66 | 0.418 |
| **EELV/PBW** |  |  |  |  |  |
| *Main Effect^†^* | Group | 6.46 | 2.50 | 6.39 | 0.011^*^ |
|  | Time | 1.16 | 0.61 | 3.63 | 0.057 |
|  | Time (Non-survivors) | 0.73 | 0.67 | 1.23 | 0.268 |
|  | Time (Survivors) | 1.81 | 1.24 | 2.23 | 0.136 |
| *Conditional Effect* | Non-survivor - Survivor (Time = 1) | -5.96 | 2.67 | -2.23 | 0.031^*^ |
|  | Non-survivor - Survivor (Time = 2) | -6.48 | 2.87 | -2.26 | 0.028^*^ |
|  | Non-survivor - Survivor (Time = 3) | -8.36 | 3.26 | -2.57 | 0.013^*^ |
|  | Time 1 - Time 2 (Non-survivors) | -0.57 | 1.24 | -0.46 | 0.892 |
|  | Time 1 - Time 3 (Non-survivors) | -1.55 | 1.73 | -0.90 | 0.647 |
|  | Time 2 - Time 3 (Non-survivors) | -0.98 | 1.74 | -0.56 | 0.841 |
|  | Time 1 - Time 2 (Survivors) | -1.09 | 1.93 | -0.57 | 0.839 |
|  | Time 1 - Time 3 (Survivors) | -3.95 | 2.09 | -1.89 | 0.159 |
|  | Time 2 - Time 3 (Survivors) | -2.86 | 2.11 | -1.36 | 0.376 |
| *Interaction Effect* | *Group × Time* | 1.18 | 1.27 | 0.91 | 0.341 |
| **EELV/preFRC** |  |  |  |  |  |
| *Main Effect* | Group | 0.128 | 0.044 | 7.96 | 0.005^**^ |
|  | Time | 0.021 | 0.0115 | 3.25 | 0.072 |
|  | Time (Non-survivors) | 0.012 | 0.013 | 0.83 | 0.362 |
|  | Time (Survivors) | 0.034 | 0.023 | 2.37 | 0.124 |
| *Conditional Effect* | Non-survivor - Survivor (Time = 1) | -0.119 | 0.047 | -2.51 | 0.016^*^ |
|  | Non-survivor - Survivor (Time = 2) | -0.124 | 0.051 | -2.42 | 0.019^*^ |
|  | Non-survivor - Survivor (Time = 3) | -0.173 | 0.059 | -2.94 | 0.005^**^ |
|  | Time 1 - Time 2 (Non-survivors) | -0.015 | 0.023 | -0.64 | 0.799 |
|  | Time 1 - Time 3 (Non-survivors) | -0.021 | 0.0322 | -0.64 | 0.800 |
|  | Time 2 - Time 3 (Non-survivors) | -0.006 | 0.033 | -0.18 | 0.983 |
|  | Time 1 - Time 2 (Survivors) | -0.020 | 0.036 | -0.56 | 0.843 |
|  | Time 1 - Time 3 (Survivors) | -0.075 | 0.039 | -1.92 | 0.151 |
|  | Time 2 - Time 3 (Survivors) | -0.055 | 0.039 | -1.39 | 0.358 |
| *Interaction Effect* | *Group × Time* | 0.025 | 0.0238 | 1.23 | 0.289 |

**Table S2.** This table compares the differences in EELV, EELV/PBW, and EELV/preFRC between the groups of survivors and non-survivors in three measurements of EELV. It also presents the differences in EELV, EELV/PBW, and EELV/preFRC at different time points among the groups. * *P* < 0.05, ** *P* < 0.01.

**Table S3.** The PaO_2_/FiO_2_ ratio, strain, Cstat, ventilatory ratio and PEEP between the groups of survivors and non-survivors at each follow-up assessment time point (1, 2 and 3) analyzed by LMM.

| Variable | Coefficient | Estimate | Std. Error | Statistic Value | *P*-value |
| --- | --- | --- | --- | --- | --- |
| **PaO_2_/FiO_2_** |  |  |  |  |  |
| *Main Effect* | Group | 99.35 | 27.40 | 11.21 | 0.001^**^ |
|  | Time | 15.53 | 11.05 | 2.00 | 0.157 |
|  | Time (Non-survivors) | 10.31 | 14.05 | 0.73 | 0.468 |
|  | Time (Survivors) | 19.67 | 19.16 | 1.13 | 0.287 |
| *Conditional Effect* | Non-survivor - Survivor (Time = 1) | -81.0 | 31.8 | -2.55 | 0.014^*^ |
|  | Non-survivor - Survivor (Time = 2) | -146.2 | 40.1 | -3.65 | 0.001^**^ |
|  | Non-survivor - Survivor (Time = 3) | -90.4 | 47.0 | -1.93 | 0.059 |
|  | Time 1 - Time 2 (Non-survivors) | -2.85 | 21.8 | -0.13 | 0.991 |
|  | Time 1 - Time 3 (Non-survivors) | -28.80 | 29.6 | -0.97 | 0.598 |
|  | Time 2 - Time 3 (Non-survivors) | -25.95 | 30.5 | -0.85 | 0.675 |
|  | Time 1 - Time 2 (Survivors) | -68.07 | 36.7 | -1.86 | 0.165 |
|  | Time 1 - Time 3 (Survivors) | -38.27 | 39.3 | -0.97 | 0.598 |
|  | Time 2 - Time 3 (Survivors) | 29.80 | 41.3 | -0.27 | 0.960 |
| *Interaction Effect* | *Group × Time* | 11.58 | 23.73 | 0.72 | 0.753 |
| **Strain** |  |  |  |  |  |
| *Main Effect* | Group | -0.072 | 0.033 | 4.59 | 0.032^*^ |
|  | Time | -0.007 | 0.009 | 0.52 | 0.473 |
|  | Time (Non-survivors) | -0.004 | 0.013 | 0.09 | 0.768 |
|  | Time (Survivors) | -0.012 | 0.013 | 0.94 | 0.332 |
| *Conditional Effect* | Non-survivor - Survivor (Time = 1) | 0.074 | 0.037 | 2.03 | 0.048^*^ |
|  | Non-survivor - Survivor (Time = 2) | 0.058 | 0.040 | 1.45 | 0.153 |
|  | Non-survivor - Survivor (Time = 3) | 0.102 | 0.048 | 2.12 | 0.038^*^ |
|  | Time 1 - Time 2 (Non-survivors) | 0.011 | 0.019 | 0.63 | 0.806 |
|  | Time 1 - Time 3 (Non-survivors) | 0.003 | 0.026 | 0.103 | 0.994 |
|  | Time 2 - Time 3 (Non-survivors) | -0.009 | 0.262 | -0.32 | 0.946 |
|  | Time 1 - Time 2 (Survivors) | -0.006 | 0.029 | -0.19 | 0.980 |
|  | Time 1 - Time 3 (Survivors) | 0.031 | 0.034 | 0.89 | 0.650 |
|  | Time 2 - Time 3 (Survivors) | 0.036 | 0.035 | 1.05 | 0.555 |
| *Interaction Effect* | *Group × Time* | -0.009 | 0.020 | 0.20 | 0.653 |
| **Cstat** |  |  |  |  |  |
| *Main Effect* | Group | 3.50 | 2.87 | 0.83 | 0.360 |
|  | Time | -1.95 | 1.28 | 2.28 | 0.130 |
|  | Time (Non-survivors) | 0.23 | 2.99 | 0.01 | 0.930 |
|  | Time (Survivors) | -2.56 | 1.25 | 4.12 | 0.042^*^ |
| *Conditional Effect* | Non-survivor - Survivor (Time = 1) | -0.67 | 4.51 | -0.15 | 0.883 |
|  | Non-survivor - Survivor (Time = 2) | -6.91 | 5.28 | -1.31 | 0.196 |
|  | Non-survivor - Survivor (Time = 3) | -5.89 | 6.40 | -0.92 | 0.361 |
|  | Time 1 - Time 2 (Non-survivors) | 1.83 | 2.53 | 0.72 | 0.752 |
|  | Time 1 - Time 3 (Non-survivors) | 6.04 | 3.54 | 1.71 | 0.220 |
|  | Time 2 - Time 3 (Non-survivors) | 4.21 | 3.68 | 1.14 | 0.497 |
|  | Time 1 - Time 2 (Survivors) | -3.07 | 4.00 | -0.77 | 0.726 |
|  | Time 1 - Time 3 (Survivors) | 1.41 | 4.73 | 0.30 | 0.952 |
|  | Time 2 - Time 3 (Survivors) | 4.48 | 4.56 | 0.98 | 0.595 |
| *Interaction Effect* | *Group × Time* | 2.32 | 2.78 | 0.72 | 0.390 |
| **Ventilatory Ratio** |  |  |  |  |  |
| *Main Effect* | Group | -0.27 | 0.22 | -1.24 | 0.207 |
|  | Time | -0.47 | 0.10 | -4.70 | <0.001^***^ |
|  | Time (Non-survivors) | -0.52 | 0.12 | -4.23 | <0.001^***^ |
|  | Time (Survivors) | -0.32 | 0.15 | -2.07 | 0.043^*^ |
| *Conditional Effect* | Non-survivor - Survivor (Time = 1) | 0.33 | 0.34 | 0.96 | 0.342 |
|  | Non-survivor - Survivor (Time = 2) | 0.57 | 0.34 | 1.66 | 0.100 |
|  | Non-survivor - Survivor (Time = 3) | -0.08 | 0.34 | -0.24 | 0.810 |
|  | Time 1 - Time 2 (Non-survivors) | 0.36 | 0.23 | 1.56 | 0.270 |
|  | Time 1 - Time 3 (Non-survivors) | 1.04 | 0.23 | 4.74 | <0.001^***^ |
|  | Time 2 - Time 3 (Non-survivors) | 0.68 | 0.23 | 2.92 | 0.013* |
|  | Time 1 - Time 2 (Survivors) | 0.60 | 0.39 | 1.55 | 0.276 |
|  | Time 1 - Time 3 (Survivors) | 0.64 | 0.39 | 1.63 | 0.240 |
|  | Time 2 - Time 3 (Survivors) | 0.03 | 0.39 | 0.08 | 0.996 |
| *Interaction Effect* | Group × Time | 0.20 | 0.23 | 0.90 | 0.363 |
| **PEEP** |  |  |  |  |  |
| *Main Effect* | Group | 0.75 | 1.41 | 0.58 | 0.570 |
|  | Time | -2.65 | 0.62 | -4.25 | <0.001^***^ |
|  | Time (Non-survivors) | -2.84 | 0.63 | -4.49 | <0.001^***^ |
|  | Time (Survivors) | -2.10 | 1.60 | -1.31 | 0.205 |
| *Conditional Effect* | Non-survivor - Survivor (Time = 1) | 0.53 | 1.08 | 0.254 | 0.800 |
|  | Non-survivor - Survivor (Time = 2) | -1.81 | 2.08 | -0.87 | 0.385 |
|  | Non-survivor - Survivor (Time = 3) | -0.95 | 2.08 | -0.46 | 0.649 |
|  | Time 1 - Time 2 (Non-survivors) | 1.64 | 1.45 | 1.13 | 0.498 |
|  | Time 1 - Time 3 (Non-survivors) | 5.68 | 1.45 | 3.91 | <0.001^***^ |
|  | Time 2 - Time 3 (Non-survivors) | 4.04 | 1.45 | 2.78 | 0.019^*^ |
|  | Time 1 - Time 2 (Survivors) | -0.70 | 2.43 | -0.29 | 0.955 |
|  | Time 1 - Time 3 (Survivors) | 4.20 | 2.43 | 1.73 | 0.201 |
|  | Time 2 - Time 3 (Survivors) | 4.90 | 2.43 | 2.02 | 0.115 |
| *Interaction Effect* | Group × Time | 0.74 | 1.42 | 0.52 | 0.605 |

**Table S3.** This table compares the differences in PaO2/FiO2, strain, Cstat, ventilatory ratio and PEEP between the groups of survivors and non-survivors in three measurements of EELV. It also presents the differences in PaO2/FiO2, strain, Cstat, ventilatory ratio and PEEP at different time points among the groups. * *P* < 0.05, ** *P* < 0.01, *** *P*<0.001.

**Table S4.** Total lung volume, total lesion volume and residual volume between the groups of survivors and non-survivors at each follow-up assessment time point (1, 2 and 3) analyzed by LMM.

| Variable | Coefficient | Estimate | Std. Error | Statistic Value | *P*-value |
| --- | --- | --- | --- | --- | --- |
| **Total Lung Volume** | |  |  |  |  |
| *Main Effect* | Group | 116.42 | 402.59 | 0.09 | 0.765 |
|  | Time | -33.83 | 108.27 | 0.10 | 0.751 |
|  | Time (Non-survivors) | -50.60 | 157.71 | 0.11 | 0.739 |
|  | Time (Survivors) | -8.32 | 148.90 | 0.00 | 0.925 |
| *Conditional Effect* | Survivor - Non-Survivor (Time = 1) | 59.4 | 419 | 0.14 | 0.888 |
|  | Survivor - Non-Survivor (Time = 2) | -664.3 | 488 | -1.36 | 0.180 |
|  | Survivor - Non-Survivor (Time = 3) | 327.8 | 546 | 0.60 | 0.551 |
|  | Time 1 - Time 2 (Non-survivors) | 378 | 239 | 1.58 | 0.274 |
|  | Time 1 - Time 3 (Non-survivors) | -131 | 336 | -0.39 | 0.920 |
|  | Time 2 - Time 3 (Non-survivors) | -510 | 354 | -1.44 | 0.339 |
|  | Time 1 - Time 2 (Survivors) | -345 | 343 | -1.01 | 0.582 |
|  | Time 1 - Time 3 (Survivors) | 137 | 343 | 0.401 | 0.916 |
|  | Time 2 - Time 3 (Survivors) | 482 | 441 | 1.10 | 0.529 |
| *Interaction Effect* | *Status×Time* | 48.37 | 211.69 | 0.05 | 0.818 |
| **Total Lesion Volume** | |  |  |  |  |
| *Main Effect* | Status | -481.62 | 213.68 | 4.92 | 0.027^*^ |
|  | Time | -96.98 | 94.92 | 0.88 | 0.348 |
|  | Time (Non-survivors) | 63.34 | 121.90 | 0.26 | 0.609 |
|  | Time (Survivors) | -301.21 | 142.77 | 3.91 | 0.048^*^ |
| *Conditional Effect* | Survivor - Non-Survivor (Time = 1) | 249 | 253 | 0.98 | 0.331 |
|  | Survivor - Non-Survivor (Time = 2) | 631 | 334 | 1.89 | 0.065 |
|  | Survivor - Non-Survivor (Time = 3) | 1014 | 391 | 2.59 | 0.013^*^ |
|  | Time 1 - Time 2 (Non-survivors) | 72.8 | 199 | 0.37 | 0.939 |
|  | Time 1 - Time 3 (Non-survivors) | -214.1 | 283 | -0.76 | 0.732 |
|  | Time 2 - Time 3 (Non-survivors) | -286.9 | 296 | -0.97 | 0.600 |
|  | Time 1 - Time 2 (Survivors) | 454.6 | 313 | 1.454 | 0.332 |
|  | Time 1 - Time 3 (Survivors) | 553.4 | 313 | 1.76 | 0.206 |
|  | Time 2 - Time 3 (Survivors) | 96.8 | 391 | 0.25 | 0.967 |
| *Interaction Effect* | *Status×Time* | -351.01 | 188.25 | 3.64 | 0.056 |
| **Residual Volume** |  |  |  |  |  |
| *Main Effect* | Status | 580.11 | 396.41 | 2.21 | 0.137 |
|  | Time | 111.42 | 122.12 | 0.82 | 0.365 |
|  | Time (Non-survivors) | -34.56 | 118.75 | 0.08 | 0.772 |
|  | Time (Survivors) | 296.62 | 239.86 | 1.58 | 0.208 |
| *Conditional Effect* | Survivor - Non-Survivor (Time = 1) | -212 | 422 | -0.50 | 0.618 |
|  | Survivor - Non-Survivor (Time = 2) | -1266 | 503 | -2.52 | 0.016^*^ |
|  | Survivor - Non-Survivor(Time = 3) | -552 | 569 | -0.97 | 0.336 |
|  | Time 1 - Time 2 (Non-survivors) | 295.9 | 259 | 1.14 | 0.499 |
|  | Time 1 - Time 3 (Non-survivors) | -94.4 | 365 | -0.26 | 0.964 |
|  | Time 2 - Time 3 (Non-survivors) | -390.3 | 384 | -1.02 | 0.574 |
|  | Time 1 - Time 2 (Survivors) | -758.0 | 376 | -2.02 | 0.138 |
|  | Time 1 - Time 3 (Survivors) | -435.9 | 376 | -1.16 | 0.492 |
|  | Time 2 - Time 3 (Survivors) | 322.1 | 482 | 0.67 | 0.785 |
| *Interaction Effect* | *Status×Time* | 340.59 | 242.22 | 2.10 | 0.147 |

**Table S4.** This table compares the differences in total lung volume, total lesion volume, and residual volume analyzed by AI software between the groups of survivors and non-survivors in three measurements of EELV. It also presents the differences in total lung volume, total lesion volume, and residual volume at different time points among the groups. * *P* < 0.05, ** *P* < 0.01.

**Table S5.** Comparation of the EELV, EELV/ PBW and EELV/preFRC between the groups of survivors and non-survivors at three time points.

| Variable | Total | Survivors | Non-survivors | β | *P*-value |
| --- | --- | --- | --- | --- | --- |
| EELV (ml) | 1287(912,1555) | 1455(1133,1995) | 1162(882,1400) | 303.03 | 0.049^*^ |
| 1 | 1281(901.2,1547.25) | 1336(1133,1732) | 1184(875,1394) | -273 | 0.105 |
| 2 | 1162(856.5,1517) | 1328(906,1952) | 1129(881,1385) | -316 | 0.081 |
| 3 | 1565(1216.75,1883.75) | 1793(1763,2340) | 1364(1017,1620) | -391 | 0.057 |
| EELV/PBW(ml/kg) | 19.96(14.63,24.46) | 24.1(20.2,35.4) | 18.5(13.4,22.7) | 6.46 | 0.011^*^ |
| 1 | 19.73(14.34,23.73) | 22.9(20.3,24.3) | 17.2(13.5,21.5) | -5.96 | 0.031^*^ |
| 2 | 19.24(13.59,23.30) | 21.2(16.8,33.9) | 18.8(12.8,22.8) | -6.48 | 0.028^*^ |
| 3 | 25.01(19.31,28.45) | 26.7(25.9,40.2) | 19.6(17.4,23.4) | -8.36 | 0.013^*^ |
| EELV/preFRC | 0.37(0.31,0.46) | 0.45(0.37,0.65) | 0.34(0.25,0.44) | 0.128 | 0.005^**^ |
| 1 | 0.36(0.29,0.45) | 0.422(0.368,0.453) | 0.324(0.250,0.435) | -0.119 | 0.016^*^ |
| 2 | 0.34(0.33,0.44) | 0.403(0.339,0.639) | 0.339(0.310,0.418) | -0.124 | 0.019^*^ |
| 3 | 0.47(0.36,0.51) | 0.493(0.491,0.708) | 0.367(0.331,0.435) | -0.173 | 0.005^**^ |
| PaO2/FiO2 (mmHg) | 168.5(125.5,261.3) | 248(202,381) | 154(109,204) | 99.35 | 0.001^**^ |
| 1 | 161(125.5,240.8) | 228(177,280) | 153(106,210) | -81 | 0.014^*^ |
| 2 | 165(119,289.5) | 382(218,393) | 135(102,180) | -146.2 | 0.001^**^ |
| 3 | 204(169,316) | 316(231,383) | 172(168,201) | -90.4 | 0.059 |
| Strain | 0.30(0.25,0.40) | 0.25(0.21,0.32) | 0.31(0.27,0.41) | -0.072 | 0.032^*^ |
| 1 | 0.31(0.27,0.41) | 0.29(0.23,0.32) | 0.33(0.27,0.42) | 0.074 | 0.048^*^ |
| 2 | 0.33(0.26,0.40) | 0.29(0.21,0.34) | 0.35(0.28,0.40) | 0.058 | 0.153 |
| 3 | 0.31(0.27,0.41) | 0.29(0.23,0.32) | 0.33(0.27,0.42) | 0.102 | 0.038^*^ |
| Cstat (ml/cmH_2_O) | 31(21,41) | 33(23,45) | 29(20,38) | -1.95 | 0.782 |
| 1 | 31(22,39) | 33(22.2,40.8) | 30(22.5,37) | -0.18 | 0.984 |
| 2 | 31(21,43) | 38(28.8 ,46.5) | 28(20.8,40.2) | 2.56 | 0.812 |
| 3 | 29(17,39) | 36.5(26,46) | 24(17,32.5) | 6.64 | 0.629 |
| Total Lung Volume(ml) | 2651(1997,3485) | 2599(1650,3442) | 2651(2029,3492) | 116.42 | 0.765 |
| 1 | 2740(2228,3507) | 2599(2083,3384) | 2975(2228,3507) | 59.4 | 0.888 |
| 2 | 2170(1631,2896) | 2527(1595,3754) | 2171(1785,2850) | -664.3 | 0.180 |
| 3 | 3402(2148,3884) | 2711(1870,3425) | 3884(2447,4060) | 327.8 | 0.551 |
| Total Lesion Volume(ml) | 1208(660,1456) | 634(467,1165) | 1313(1058,1634) | -481.62 | 0.027^*^ |
| 1 | 126.2(673.5,1442.3) | 968(568,1526) | 1313(1098,1442) | 249 | 0.331 |
| 2 | 1053(651,1406) | 796(452,951) | 1313(897,1672) | 631 | 0.065 |
| 3 | 1133(529,1596) | 465(382,680) | 1596(1233,2164) | 1014 | 0.013^*^ |
| Residual Difference Volume(ml) | 1292(564,2153) | 1456(644,2918) | 1278(564,1925) | 580.11 | 0.137 |
| 1 | 1328.9(684.2,2329.3) | 1078(285,2619) | 1478(762,2036) | -212 | 0.618 |
| 2 | 682.7(463.9,1624.6) | 1758(644,3302) | 628(388,1560) | -1266 | 0.016^*^ |
| 3 | 1802.4(1214.2,2269) | 1944(1437,2494) | 1802(1214,1904) | -552 | 0.336 |

**Table S5.** This table presents the comparison results between the groups of survivors and non-survivors for all variables, displayed using medians along with interquartile ranges. * *P* < 0.05, ** *P* < 0.01.
